# Analysis of a multicentre cloud-based CT dosimetric database: preliminary results

**DOI:** 10.1186/s41747-019-0105-6

**Published:** 2019-07-16

**Authors:** Francesca Calderoni, Federica Campanaro, Paola Enrica Colombo, Mauro Campoleoni, Cristina De Mattia, Federica Rottoli, Giannicola Galetta, Fabio Zucconi, Andrea Pola, Andrea Righini, Fabio Triulzi, Angelo Vanzulli, Alberto Torresin

**Affiliations:** 1 Department of Medical Physics, ASST Grande Ospedale Metropolitano Niguarda, Piazza Ospedale Maggiore 3, 20162 Milan, Italy; 2 Medical Physics Unit, Fondazione IRCCS Ca’ Granda Ospedale Maggiore Policlinico, Via Pace 9, 20122 Milan, Italy; 3 Medical Physics, ASST Fatebenefratelli Sacco, via G.B. Grassi 74, 20157 Milan, Italy; 4 Department of Energy, Politecnico di Milano, via La Masa 34, 20156 Milan, Italy; 5 Pediatric Radiology and Neuroradiology Unit, Children’s Hospital V. Buzzi, Via Castelvetro 32, 20154 Milan, Italy; 6 Department of Neuroradiology, Fondazione IRCCS Ca’ Granda Ospedale Maggiore Policlinico, Via Pace 9, 20122 Milan, Italy; 7 Department of Radiology, ASST Grande Ospedale Metropolitano Niguarda, Piazza Ospedale Maggiore 3, 20162 Milan, Italy

**Keywords:** Radiation dosage, Radiation exposure, Software, Tomography scanners (x-ray computed), Radiation dose index monitoring software

## Abstract

**Background:**

To manage and analyse dosimetric data provided by computed tomography (CT) scanners from four Italian hospitals.

**Methods:**

A radiation dose index monitoring (RDIM) software was used to collect anonymised exams stored in a cloud server. Since hospitals use different names for the same procedure, digital imaging and communications in medicine (DICOM) tags more appropriate to describe exams were selected and associated to study common names (SCNs) from a radiology playbook according to scan region and use of contrast media. Retrospective analysis was carried out to describe population and to evaluate dosimetric indexes and inaccuracies associated with SCNs.

**Results:**

More than 400 procedures were clustered into 95 SCNs, but 78% of exams on adults were described with only 10 SCNs. Median values of dose-length product (DLP) and volumetric CT dose index (CTDI_vol_) for three analysed SCNs were in agreement with those previously published. The percentage of inaccuracies does not heavily affect the dosimetric analysis on the whole cloud, since variations in median values reached at most 8%.

**Conclusions:**

Implementation of a cloud-based RDIM software and related issues were described, showing the strength of the chosen playbook-based clustering and its usefulness for homogeneous data analysis. This approach may allow for optimisation actions, accurate assessment of the risk associated with radiation exposure, comparison of different facilities, and, last but not least, collection of information for the implementation of the 2013/59 Euratom Directive.

## Key points

- A radiation dose index monitoring software allowed to collect data on radiological exams and to store them in a cloud server.
- Clustering examinations through a radiological playbook is a good choice for data analysis.
- More than 400 computed tomography procedures were clustered into 95 study common names.
- Dose indexes for analysed study common names agree with those previously published.
- The inaccuracies of the system did not heavily affect dosimetric analysis on the whole cloud.

## Background

The extensive use of computed tomography (CT) examinations in radiological diagnostics [1] caused an increasing attention to patient exposure and to the potential risk of carcinogenesis associated with relatively high radiation doses. Optimisation is mandatory to maintain the quality of the diagnostic information provided by the examination while seeking to reduce patient exposure to radiation to a level as low as reasonably achievable. The International Commission on radiological protection (ICRP) stated that a further optimisation can be obtained through collection of data from radiation dose structured reports in a digital format and through electronic data transfer from hospital and radiology information systems, providing data for large numbers of patients suitable for collection in a registry [2].

Hence, a useful way to monitor ionising radiation exposure caused by radiologic examinations is the adoption of a radiation dose index monitoring (RDIM) software. These software packages also allow to verify the compliance with the diagnostic reference levels (DRLs), facilitating surveys and improving the statistical strength of the analysis [3–5], optimise the exposures and compare different protocols or scanners. In order to analyse the exams performed with different CT scanners and in different hospitals in a consistent way, it is also necessary to cluster such large amount of data.

A great collection of data with these aims has been performed since 2011 by the American College of Radiology (ACR) that created the Dose Index Registry (DIR) [6]. The DIR includes more than 50 million CT exams transmitted automatically from scanners and arranged according to the RadLex® playbook by the Radiological Society of North America [7, 8]. Kanal et al. [9] analysed the ten most common examinations within the DIR in order to develop DRLs and achievable doses as a function of patient size, obtaining values similar to those obtained by other countries for median-size patients. One of the limitations they highlighted are the unavoidable inaccuracies in examination clustering that may cause problems both in estimation of benchmark data and in comparison with them.

Parakh et al. [10] presented their experience with a radiation tracking software (RTS) for monitoring and comparing in relative terms cumulative patient effective doses and for calculating the average dose metrics, hence providing a global view of CT doses and defining a meaningful benchmark representing institutional DRLs. They stated that a critical step was to ensure that CT protocols on all scanners were consistently identified, a goal achieved by adopting the RadLex® playbook.

Another publication by Parakh et al. [11] extended this kind of work to six medical institutions by collecting anonymised data from local servers into a single master server. The RTS allowed to perform analysis of different dose metrics, *i.e*., volumetric CT dose index (CTDI_vol_), dose-length product (DLP) and size-specific dose estimate and effective dose. To ensure a consistent analysis, a great effort was exerted in protocol matching using the RadLex® playbook. Furthermore, the large number of CT scans reduced the effect of erroneous cases on the average dose metrics.

Also, Pyfferoen et al. [12] collected anonymised data from several hospitals through a RTS. They grouped the different protocol names under the reference anatomical regions according to available national DRLs in order to compare dose levels and scan lengths of standard adult CT examinations within three institutions and with national reference levels. Before the analysis, they performed a data check to eliminate, on the series level, those examinations in which the CT region did not match the clinical indication. Data checking at series level was performed also by MacGregor et al. [13] to verify the belonging to specific “master protocols” used for the clustering.

In addition to commercial systems, Boos et al. [14] implemented an in-house cloud-based CT RTS to automatically monitor dose data to make a comparison with national DRLs. Even if this study reported initial single-centre results, the cloud-based approach enabled multicentre applications.

A cloud-based RDIM software was chosen by our group within a research project endorsed by Regione Lombardia, Italy. One of the aims of this project was to manage and analyse dosimetric data. A relevant task was to create a central database of dosimetric data, analysing exposure values collected through 13 CT scanners installed in four different hospitals. The goal of this paper is to describe the feasibility of the cloud solution, presenting some preliminary results and discussing advantages and disadvantages of this cloud-based system.

## Methods

The study was evaluated by our Institutional Review Board, and the requirement for informed consent was waived. The four hospitals involved were as follows: ASST Grande Ospedale Metropolitano Niguarda, Fondazione IRCCS Ca’ Granda Ospedale Maggiore Policlinico, ASST Fatebenefratelli Sacco and Ospedale dei Bambini V. Buzzi. The first three are general hospitals, while the last one is paediatric.

To analyse the dosimetric archive, the associated large amount of data was clustered according to the RadLex® playbook [15] as in the previously cited papers [9–11].

Data collected in 2017 were first analysed according to facilities, age and sex, to get descriptive statistics. In a second step, the distributions of dosimetric quantities were compared with values from the literature to check the strength of the cloud database. A systematic comparison with currently available reference levels was beyond the scope of this work. The same data were also used to assess the quantity of studies with series not matching with the original requirement and to evaluate their effect on dosimetric quantities.

### Description of the RDIM software and cloud server architecture

The four hospitals were equipped with the RDIM software Bracco Injeneering’s NEXO [DOSE]® (Bracco Injeneering S.A., Lausanne, Switzerland), developed by PACSHealth, LLC, integrated with the different PACS of each hospital (Agfa, Fuji, Carestream). NEXO [DOSE]® is a web-based software which collects patient information (age, sex, etc.) and dosimetric data.

Relevant data could be extracted from different sources: digital imaging and communications in medicine (DICOM) header (for each series), patient protocol (overall exam) and radiation dose structured report. Dealing with several CT scanners, the most suitable data sources were chosen for each device.

Data from the hospitals were collected both in local servers and in a cloud one.

Each individual institution has complied with its internal procedures to ensure the highest level of security and privacy of patient personal data. These procedures required the appointment of a person in charge of data processing, in this case, an external subject, the specification of access methods and the definition of the persons authorised to access the data, who undertake to behave in absolute confidentiality. In the case of the cloud server, data were anonymised prior to leaving the local site server following DICOM PS3.15 [16] and Integrating the Healthcare Enterprise Radiation Exposure Monitoring RAD-63 profiles [17]. Patient data were removed, replaced, or modified in accordance with the reference DICOM standard. There were some exceptions, such as patient characteristics (age, sex, height, weight) and device information (facility, device, exposure parameters), for relevant data needed for statistical aims and analysis. Only the relevant data were transmitted, not the entire studies.

Finally, all data were collected in the Cloud NEXO [DOSE]® Server – Microsoft Azure for Healthcare, in compliance with Health Insurance Portability and Accountability Act, International Organization for Standardization and European Union data protection directives, received via DICOM over transport layer security (TLS).

Each hospital could access the cloud database, and the software was able to report, for each exam, patient demographics (age, sex), and scan protocol information (CTDI_vol_, DLP), with all the previously anonymised sensitive data. More detailed information relative to the single series could also be retrieved.

### Global descriptive analysis

The analysis of the whole data stored in the cloud server can give a general description of the distribution of radiological exams among the population depending on facilities, age and sex.

Through NEXO [DOSE]®, data were filtered according to facility, device, age, sex, and other characteristics in order to develop descriptive statistics of parameters relevant for the risk associated to radiation exposure, such as the percentages of males and females undergoing exams and the distribution of the number of patients as a function of age. Moreover, the number of exams of the different hospitals was tracked.

A first retrospective analysis regarding CT exams performed during 2017 was carried out.

### Detailed analysis of CT studies

#### Clustering

The RadLex® playbook is a project of the Radiological Society of North America [8] that provides a standardised system for naming radiological procedures. As in other studies [9–11], the RadLex® playbook was used to cluster a great quantity of data for the subsequent analysis.

The arrangement of exams in homogeneous groups, according to scan region and acquisition task, is difficult due to the differences in types of CT scanner, radiology information systems (RIS) and picture archiving and communication system (PACS). We solved this problem using the radiological information stored in the different DICOM tags and inside the hospital reporting database. In two hospitals, the DICOM tag “study description” (0008,1030) generated by the RIS was used; in another hospital, the same tag generated by the scanner was considered, whereas in the last one, the DICOM tag “protocol name” (0018,1030) compiled with the scanner protocol name was chosen.

**Fig. 1:**
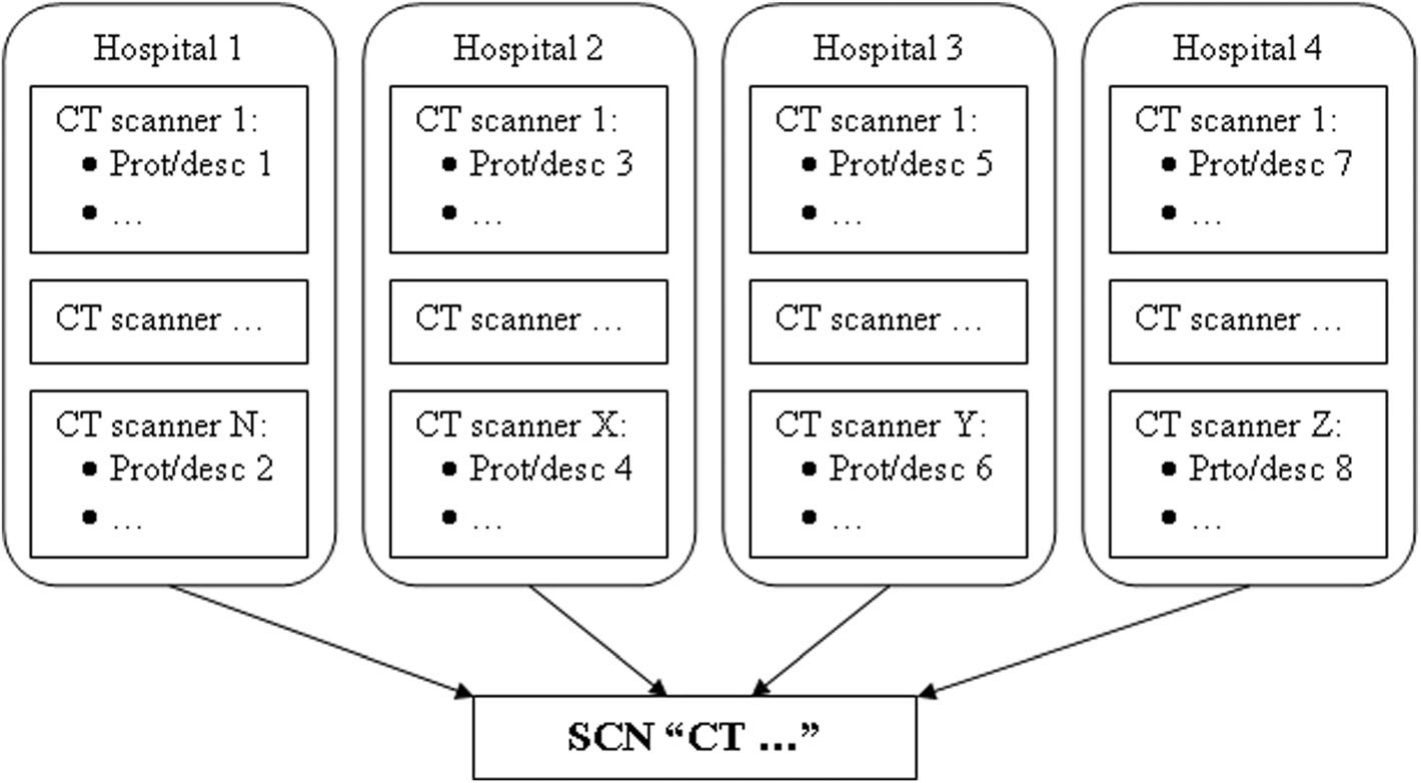
Association of different protocol names or descriptions (Prot/desc) to study common names (SCNs)

A study common name (SCN) from the RadLex® playbook was associated to each description or protocol name to classify exams in a consistent way, according to scan region and use of contrast media, as represented in general terms in Fig. 1. Since the procedures within a RadLex® label should have homogeneous exposure parameters, data organised in this way were used to analyse population and dosimetric quantities in a consistent way in order to evaluate the different radiological procedures.

Through NEXO [DOSE]®, data were filtered according to SCN and both studies and series were exported in Excel format. Files relative to studies include mean CTDI_vol_ and total DLP, allowing to evaluate their distributions and to calculate median values, 25th and 75th percentiles. Variations of DLP with sex were also evaluated.

In the first phase of the study, 76,171 exams on adult patients (age range 18–109 years), the most frequent ones without administration of contrast agent (without contrast, “WO”) were considered, in particular, those belonging to SCNs “CT Head WO” (35%, 27,030 exams), “CT Chest WO” (9%, 6,635 exams), and “CT Abd/Pelv WO” (2%, 1,778 exams) in order to evaluate radiation exposure in different body regions. This analysis was performed at first with the whole data in the cloud, excluding those studies with coarse problems in the data transfer from the DICOM tag. In the case of “CT Chest WO” and “CT Abd/Pelv WO”, only data from three hospitals were analysed since the few studies of the fourth one were not enough for statistical aims. CTDI_vol_ and DLP values were depicted through histograms, including in the highest class values higher than the depicted range, but to check the strength of this clustering, only median values of dosimetric quantities were compared with recent DRLs, as indicated by ICRP 135 [2].

#### Check of clustered data

In the second phase of the study, a more accurate check of data within the SCNs was performed through series analysis following the methodology proposed in other publications [12, 13]. Studies including series not in agreement with the SCN in terms of anatomical region or use of contrast media (exams not in line with the SCN) were quantified and removed from the subsequent analysis. For example, within the SCN “CT Head WO” studies including series descriptions as “TorAdd 3.0 B40f”, “C_Spine”, “HeadAngio 0.75 H30f”, and “Spine 2.0 B30s” were found and removed; the same for description as “HeadSeq 4.8 H31s” within the SCN “CT Chest WO”.

The analysis of CTDI_vol_ and DLP was thus repeated only with the studies in line with the SCNs in order to evaluate potential changes in median values and to test the strength of the database.

## Results

### Global descriptive analysis

**Fig. 2:**
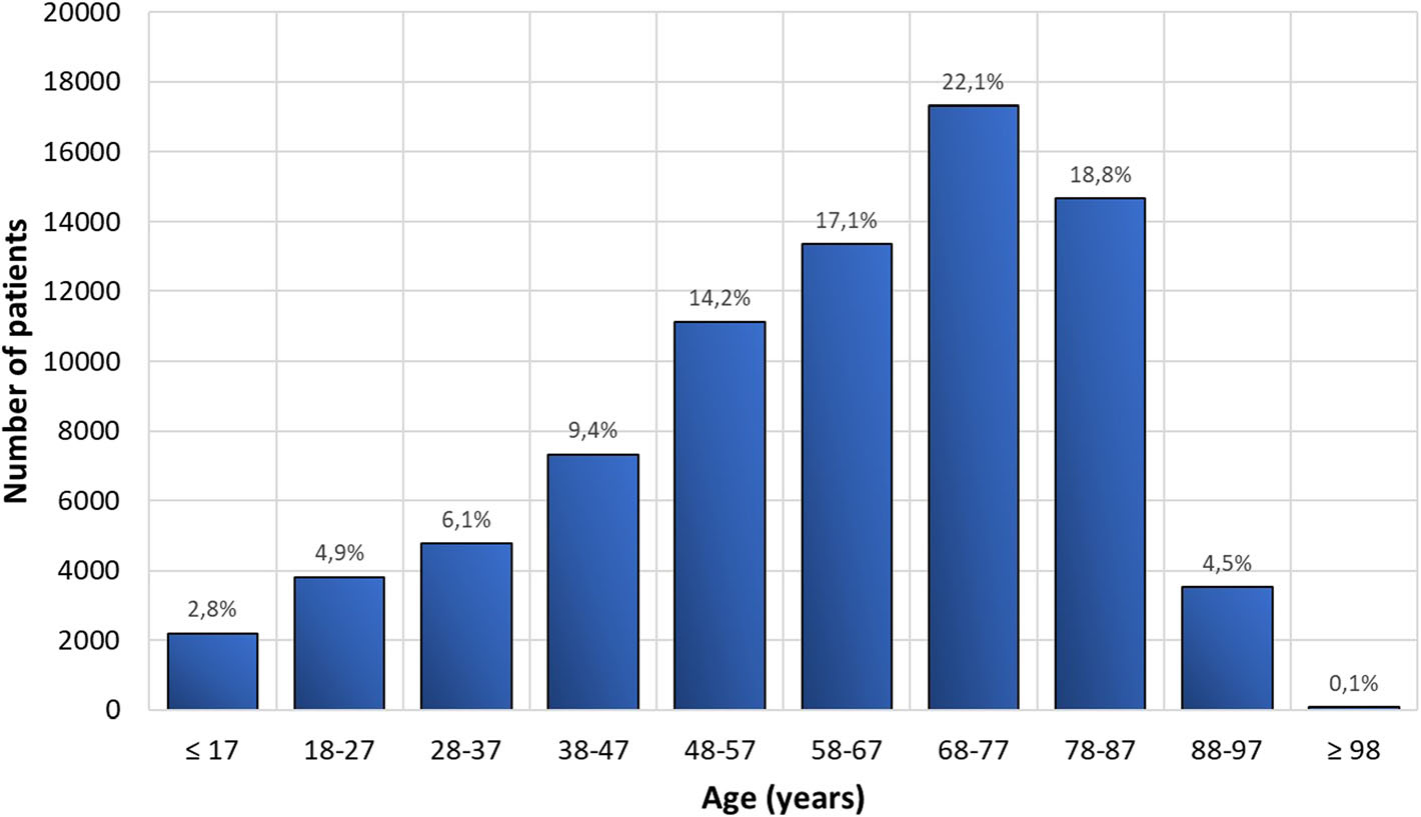
Age distribution of patients who had computed tomography in 2017

All the 78,370 exams, including paediatric and adult ones, performed in the four hospitals were analysed. In particular, 49% of the exams were carried out at hospital 1, 15% at hospital 2, 34% at hospital 3, and 2% at hospital 4. The age distribution of the whole CT examinations is shown in Fig. 2. The large majority of exams are performed on adult patients (≥ 18 years old), 97.2% against 2.8% of paediatrics (< 18 years old), with predominance in the 68–77 years old range. The distribution in terms of sex was as follows: 53.4% of patients were male whilst 46.6% were female.

### Detailed analysis of studies

#### Clustering

**Fig. 3:**
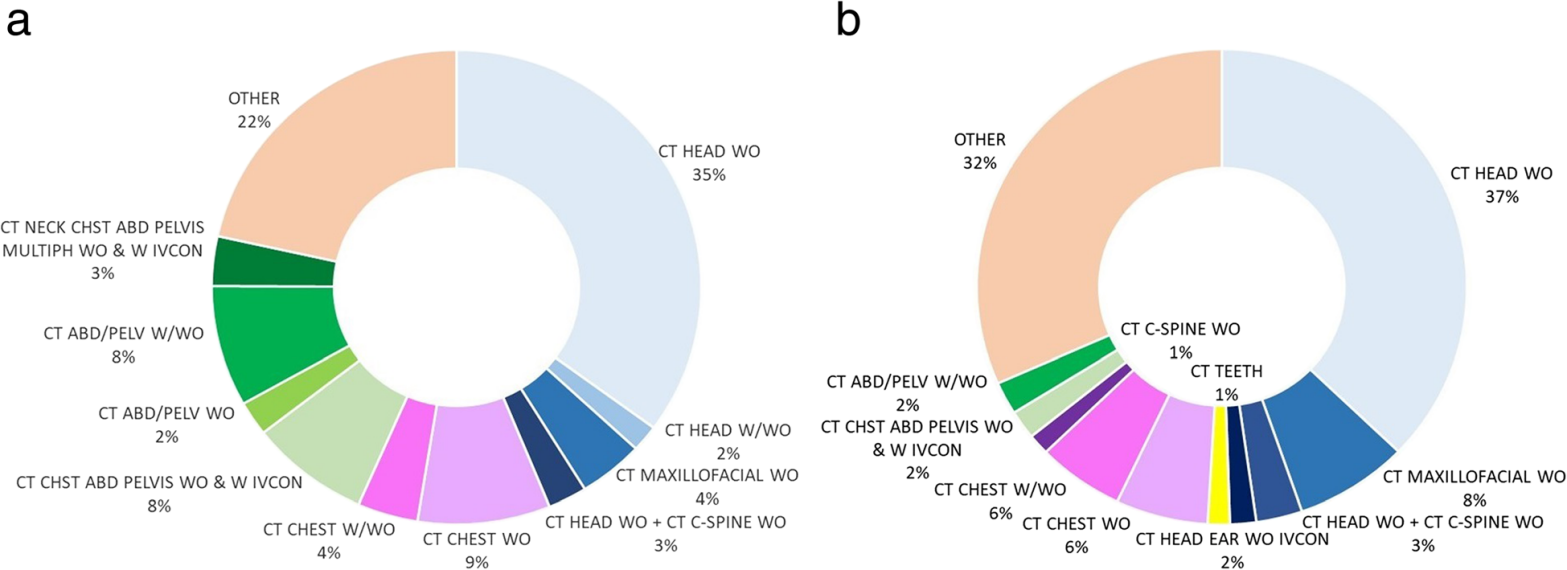
Percentages of exams within the ten most common study common names in two different age ranges: 18–109 (**a**) and 0–17 (**b**). The main differences between the two distributions are the presence of many exams with a broad scan length including different anatomical regions in adult pie chart and the almost total absence of exams with contrast agent in paediatric ones. The majority of paediatric exams were in the head region compared to the adult distribution, with more “CT Maxillofacial WO” (traumas, sinusitis) and the addition of “CT Teeth”. The higher percentage of exams in the “OTHER” in the paediatric distribution could be related to the greater difficulty of the diagnosis in the absence of clinical history and with symptoms described by children

More than 400 CT procedures were clustered into 95 SCNs. Figure 3 shows the percentages of exams within the 10 most common SCNs, clustering a quantity of exams between 78% (adult) and 68% (paediatric) of the total, depicted for different age ranges: 18–109 and 0–17. It is evident that the prevalent exam is the “CT Head WO”, followed by SCNs with far fewer studies (less than 10%) such as “CT Chest WO”, “CT Abd/Pelv W/WO”, and “CT Chst Abd Pelvis WO & W IVCON”, which include all the remaining anatomical regions.

#### Results for adults: “CT Head WO”

Table 1 shows the median values of DLP and CTDI_vol_ for SCN “CT Head WO” for the different hospitals (26,965 exams over 27,030). Hospital 1 had the lowest values of both DLP and CTDI_vol_ while hospital 2 and hospital 4 had the highest values for DLP and CTDI_vol_, respectively.

**Table 1:** Dose-length product (DLP) and volumetric computed tomography dose index (CTDI_vol_) for the study common name “CT Head WO”: data from individual hospitals, total and reference values

	Number of exams/number of scanners	DLP (mGy × cm) Median (25th–75th percentile)	CTDI_vol_ (mGy) Median (25th–75th percentile)
Hospital 1	9,961/6	853 (798–898)	54.8 (53.3–57.8)
Hospital 2	5,578/2	1,131 (1,004–1,339)	60.4 (51.3–60.9)
Hospital 3	11,031/4	1,017 (830–1,022)	58.6 (54.8–58.7)
Hospital 4	395/1	1,121 (969–1,468)	68.4 (60.5–76.0)
Total	26,965/13	1,011 (827–1,024)	58.6 (53.3–58.9)
ISTISAN 17/33 [18]		1,382	69.0
RP 180 [19]		1,000 (760–1,300)*	60 (50–75)*
Canadian survey [20]		1,276 (1,084–1,463)	74.4 (60.1–79.1)

*Most common value (range)

DLP for females was lower than that for males of about 5%, mainly due to different scan lengths.

**Fig. 4:**
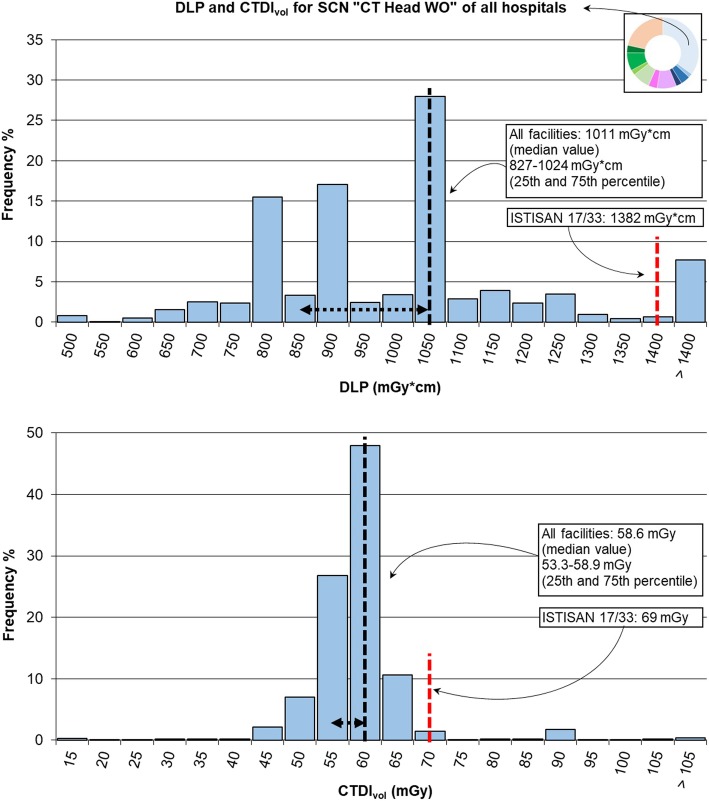
Distribution of total dose-length product (DLP) and volumetric computed tomography dose index (CTDI_vol_) for study common name (SCN) “CT Head WO” and comparison with values from ISTISAN 17/33 [18]. The median value and range between 25th and 75th percentile are shown in black dashed lines; the red dashed line reports the diagnostic reference level (DRL) provided by ISTISAN 17/33 [18]. The narrow shape of CTDIvol histogram points out similar values among scanners and highlights the use of fixed mAs values. The DLP histogram shows several peaks. On the one hand, the anatomical region was very similar from one patient to another; on the other hand, the scanners used different collimations, and therefore, the entire anatomical range is covered by a fixed and scanner-dependent number of acquisitions (axial mode). The final peak, more or less evident in all distributions (see the following Figs. 5, 6, 7, 8, and 9), is caused by the inclusion in the last class of values higher than the considered range

Figure 4 shows the distributions of DLP and CTDI_vol_ for the whole data of the SCN “CT Head WO” related to the DRL values provided by the new Italian publication ISTISAN 17/33 [18].

Median values of radiation exposure with their 25th and 75th percentiles are also summarised in Table 1 (median DLP of 1011 mGy × cm; median CTDI_vol_ of 58.6 mGy).

The total DLP distribution had a median value of 1,011 mGy × cm, close to (1.1% higher) the DRLs summarised by the 2014 European Commission Radiation Protection document 180 [19], which considers the most common value of 1,000 mGy × cm and a range of 760–1,300 mGy × cm. The same was for the total CTDI_vol_ of 58.6 mGy which is 2.3% lower than the most common value of 60 mGy with a range of 50–75 mGy.

The same comparison can be performed with values provided by recent publications on this topic, *i.e*., the US Diagnostic Reference Levels and Achievable Doses [9] and the Canadian Computed Tomography Survey [20]. Our median values of DLP and CTDI_vol_ were 5.1% and 4.6%, respectively, higher than the US DRLs (expressed as 75th percentile for a median size patient), *i.e*., 962 mGy × cm and 56 mGy, respectively. Compared to Canadian survey, our values were 20.8% and 21.2% lower, respectively, than those indicated in the subgroup “Adult head-helical/no contrast/fixed current” in terms of median value (25th percentile–75th percentile): DLP of 1,276 mGy × cm (1,084 mGy × cm–1,463 mGy × cm), CTDI_vol_ of 74.4 mGy (60.1 mGy–79.1 mGy).

#### Results for adults: “CT Chest WO”

The median values of DLP and CTDI_vol_ for SCN “CT Chest WO” are shown in Table 2 (6,542 exams over 6,635). In this case, hospital 3 has the lowest values of both the dosimetric indexes, whereas hospitals 2 and 1 have the highest values for DLP and CTDI_vol_, respectively. Variations with sex showed values of DLP for females lower than that for males at about 19%, mainly due to lower CTDI.

**Table 2:** Dose-length product (DLP) and volumetric computed tomography dose index (CTDI_vol_) for the study common name “CT Chest WO”: data from individual hospitals, total and reference values

	Number of exams/number of scanners	DLP (mGy × cm) Median (25th–75th percentile)	CTDI_vol_ (mGy) Median (25th–75th percentile)
Hospital 1	3,513/5	268 (194–335)	7.4 (5.9–8.5)
Hospital 2	1,003/2	323 (232–468)	7.1 (5.9–8.8)
Hospital 3	2,026/4	247 (169–345)	6.1 (4.9–8.5)
Total	6,542/11	268 (190–349)	7.0 (5.4–8.5)
ISTISAN 17/33 [18]		754	15.0
RP 180 [19]		400 (270–700)*	10 (10–30)*
Canadian survey [20]		302 (197–440)	8.5 (5.7–13.0)

*Most common value (range)

**Fig. 5:**
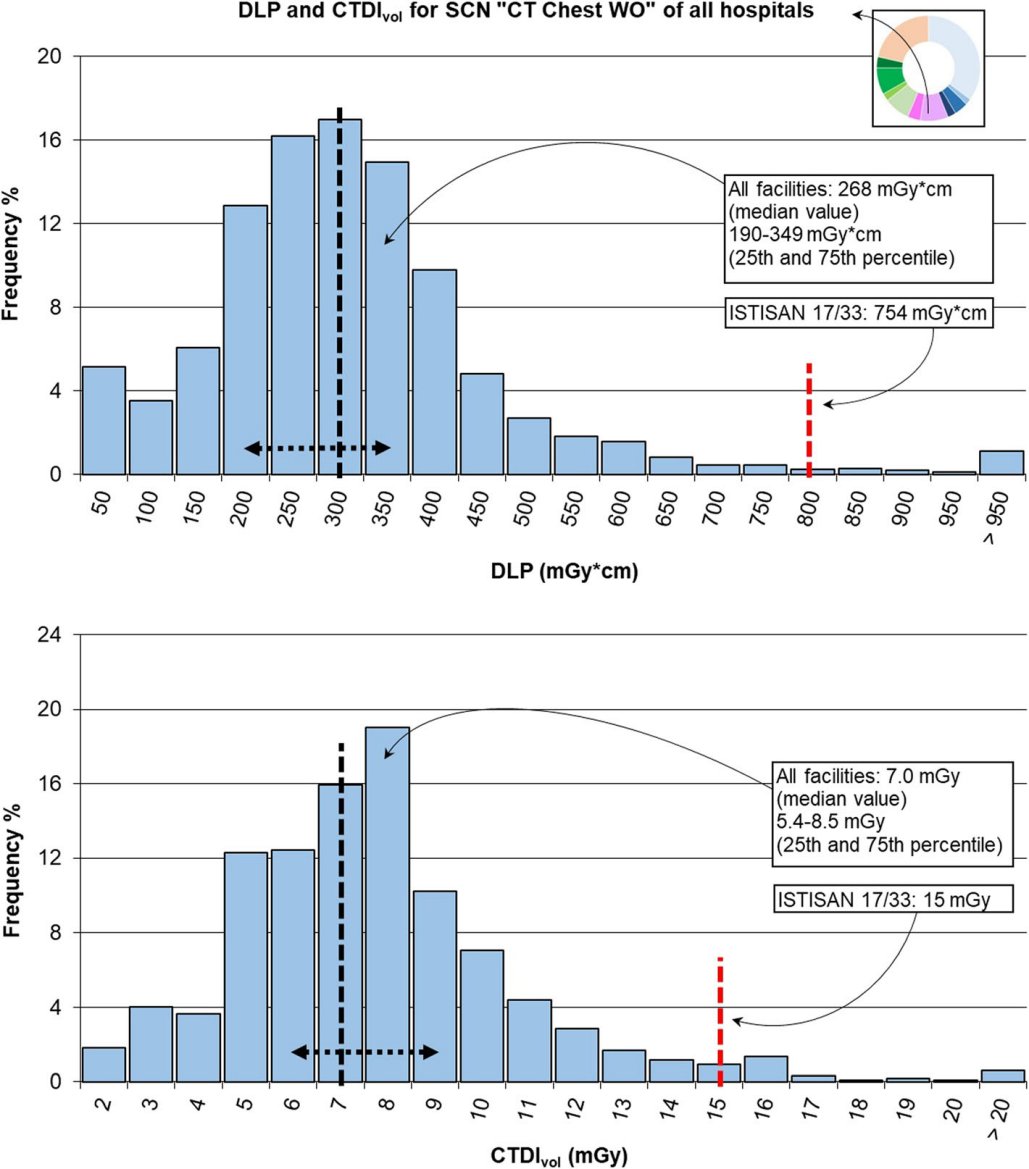
Distribution of total dose-length product (DLP) and volumetric computed tomography dose index (CTDIvol) for study common name (SCN) “CT Chest WO” and comparison with values from ISTISAN 17/33 [18]. The median value and range between 25th and 75th percentile are shown in black dashed lines; the red dashed line reports the diagnostic reference level (DRL) provided by ISTISAN 17/33 [18]

In the case of “CT chest WO” of Fig. 5, both the total DLP distribution and the CTDI_vol_ distribution have the shape of a gamma function as expected [1, 5]. The total DLP reported in Table 2 has a median value of 268 mGy × cm, 33.0% lower than the DRL provided by the RP 180 [19] which considers the most common value of 400 mGy × cm and a range of 270–700 mGy × cm. Also, in the case of the total CTDI_vol_, the median value of 7.0 mGy is 30.0% lower than the most common value of 10 mGy with a range of 10–30 mGy.

The US DRLs [9] report a DLP of 443 mGy × cm and a CTDI_vol_ of 12 mGy. Hence, our results were 39.5% and 41.7% lower, respectively. The data provided by the Canadian computed tomography survey [20] for the subgroup “Adult chest-helical/no contrast/dose reduction” were DLP 302 mGy × cm (197 mGy × cm–440 mGy × cm), CTDI_vol_ 8.5 mGy (5.7 mGy–13.0 mGy). Hence, our results were 11.3% and 17.6% lower, respectively.

#### Results for adults: “CT Abd/Pelv WO”

The median values of DLP and CTDI_vol_ for SCN “CT Abd/Pelv WO” are reported in Table 3 (1,692 exams over 1,778). As for the SCN “CT head WO”, the lowest values are those of hospital 1 while hospital 2 has again the highest values. Variations with sex show values of DLP for females lower than that for males of about 3%, due both to lower CTDI and scan length.

**Table 3:** Dose-length product (DLP) and volumetric computed tomography dose index (CTDI_vol_) for the study common name “CT Abd/Pelv WO”: data from individual hospitals, total and reference values

	Number of exams/number of scanners	DLP (mGy × cm) Median (25th–75th percentile)	CTDI_vol_ (mGy) Median (25th–75th percentile)
Hospital 1	1,081/5	543 (425–743)	10.4 (8.2–13.1)
Hospital 2	326/2	643 (539–852)	13.1 (11.5–15.6)
Hospital 3	285/4	590 (455–771)	11.8 (10.1–14.3)
Total	1,692/11	569 (446–769)	11.2 (8.9–14.0)
ISTISAN 17/33 [18]		920	15.0
Canadian survey [20]		516 (349–735)	12.9 (8.6–17.6)

**Fig. 6:**
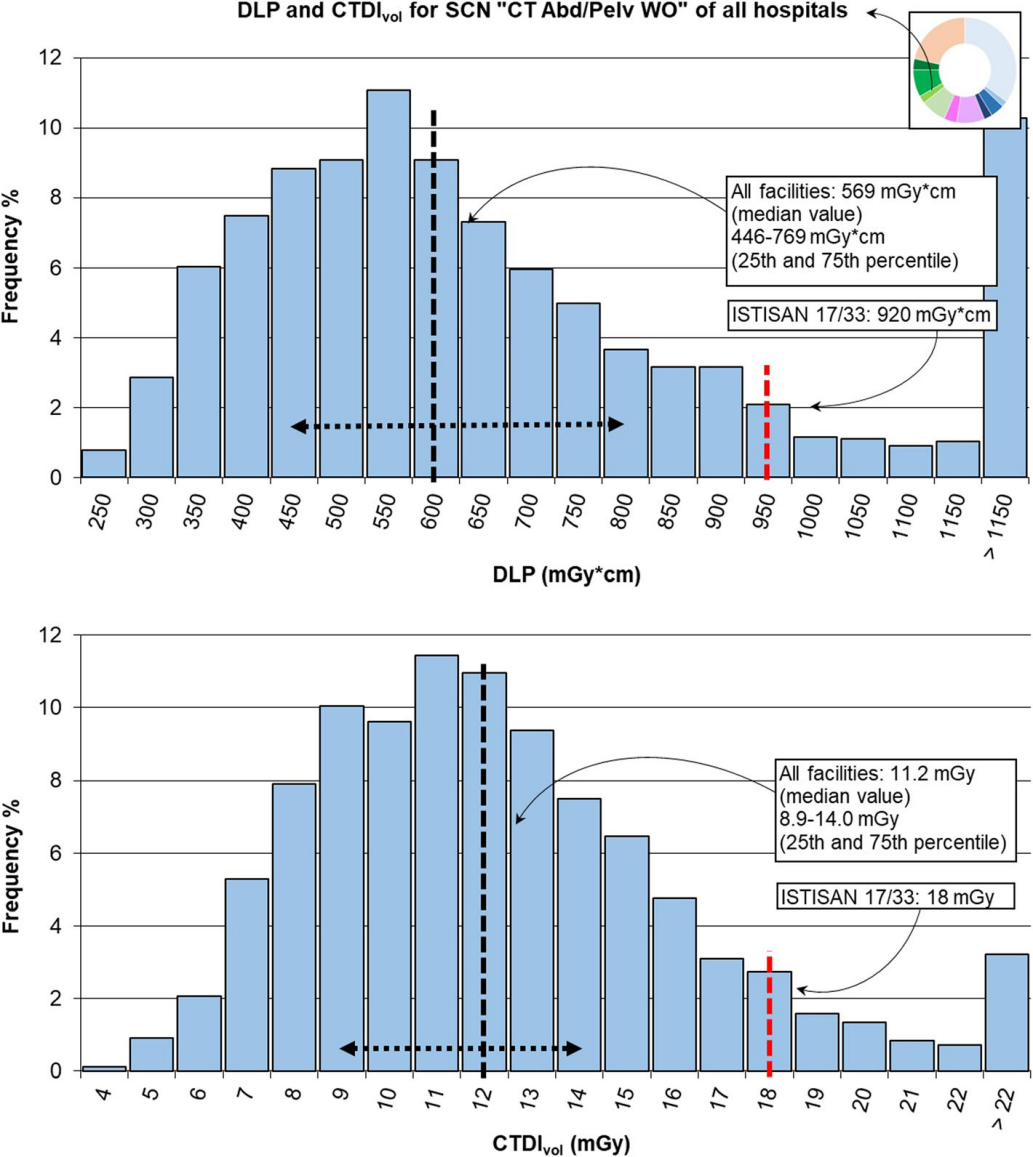
Distribution of total dose-length product (DLP) and volumetric computed tomography dose index (CTDI_vol_) for study common name (SCN) “CT Abd/Pelv WO” and comparison with values from ISTISAN 17/33 [13]. The median value and range between 25th and 75th percentile are shown in black dashed lines; the red dashed line reports the diagnostic reference level (DRL) provided by ISTISAN 17/33 [18]

The total DLP and CTDI_vol_ distributions for the SCN “CT Abd/Pelv WO” of Fig. 6 had median values of 569 mGy × cm and 11.2 mGy, respectively, summarised in Table 3.

A comparison with the values from the European Commission Radiation Protection document 180 [19] was not possible since this document provides two different values for the abdomen and pelvis. Compared to the US DRLs [9] (DLP equal to 781 mGy × cm and CTDI_vol_ to 16 mGy), our values were 27.1% and 30.0% lower, respectively. These results, DLP 10.3% higher and CTDI_vol_ 13.2% lower, are also comparable with the data obtained by the Canadian Computed Tomography Survey [20]: DLP 516 mGy × cm (349 mGy × cm–735 mGy × cm), CTDI_vol_ 12.9 mGy (8.6 mGy–17.6 mGy).

#### Check of clustered data

The series analysis shows that some irradiation events do not belong to the considered SCN. This means that the study changes compared to the prescription, but it can be justified by the necessity of more information in relation to the initial clinical question. The percentage of exposures not in line with the analysed SCNs is different for single hospitals, as summarised in Table 4 in comparison with the whole data. Considering the whole data from the four facilities, the exposures in line with the SCN are always about 90%, as reported in Table 4.

**Table 4:** Ratio and percentage of exposures not in line (*i.e.*, not in agreement with the study common name in terms of body region studied or use of contrast media) for the three analysed study common names: individual hospitals and total

	CT Head WO	CT Chest WO	CT Abd/Pelv WO
Hospital 1	167/9,961 (2%)	42/3,513 (1%)	100/1,081 (9%)
Hospital 2	1,269/5,578 (23%)	64/1,003 (6%)	29/326 (9%)
Hospital 3	277/11,031 (3%)	442/2,026 (22%)	51/285 (18%)
Hospital 4	0/395 (0%)	NA	NA
Total	1,713/26,965 (6%)	548/6,542 (8%)	180/1,692 (11%)

Data in parentheses are percentages. *NA* Not available

**Fig. 7:**
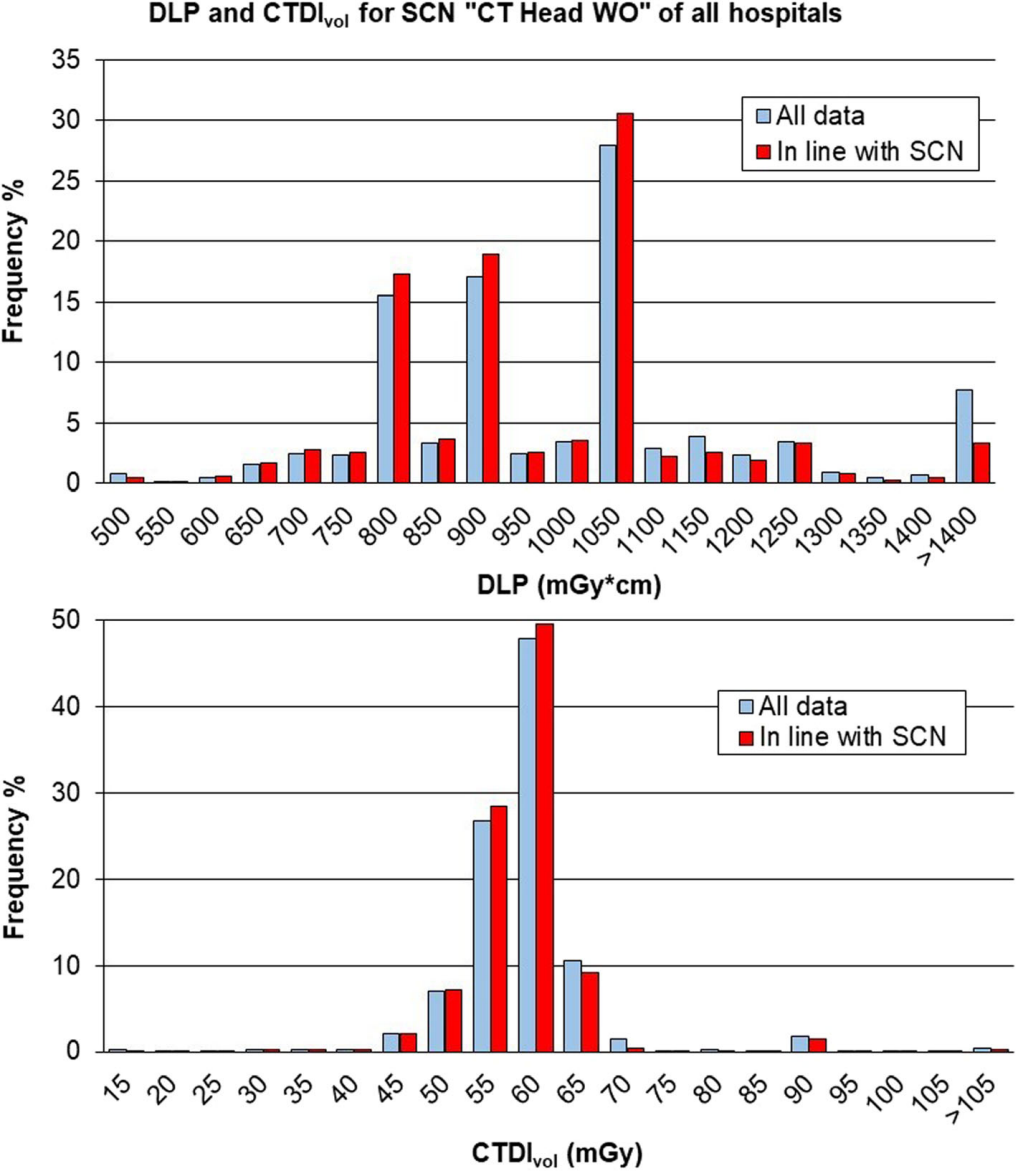
Comparison between distributions of total dose-length product (DLP) and volumetric computed tomography dose index (CTDI_vol_) of the whole data and those in line with the study common name (SCN) “CT Head WO”, *i.e*., studies with all series belonging to that cluster

**Fig. 8:**
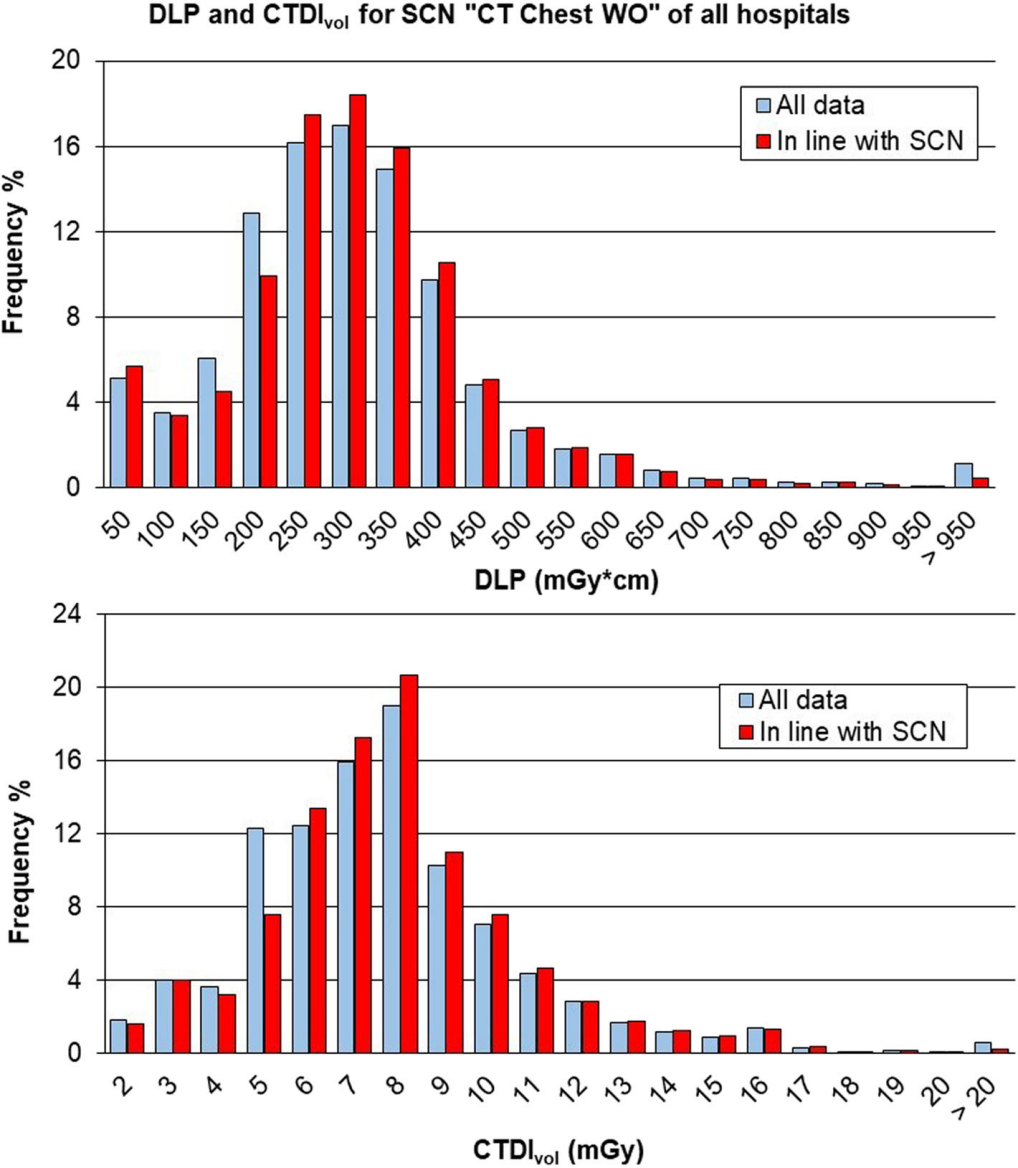
Comparison between distributions of total dose-length product (DLP) and volumetric computed tomography dose index (CTDI_vol_) of the whole data and those in line with the study common name (SCN) “CT Chest WO”, *i.e*., studies with all series belonging to that cluster

**Fig. 9:**
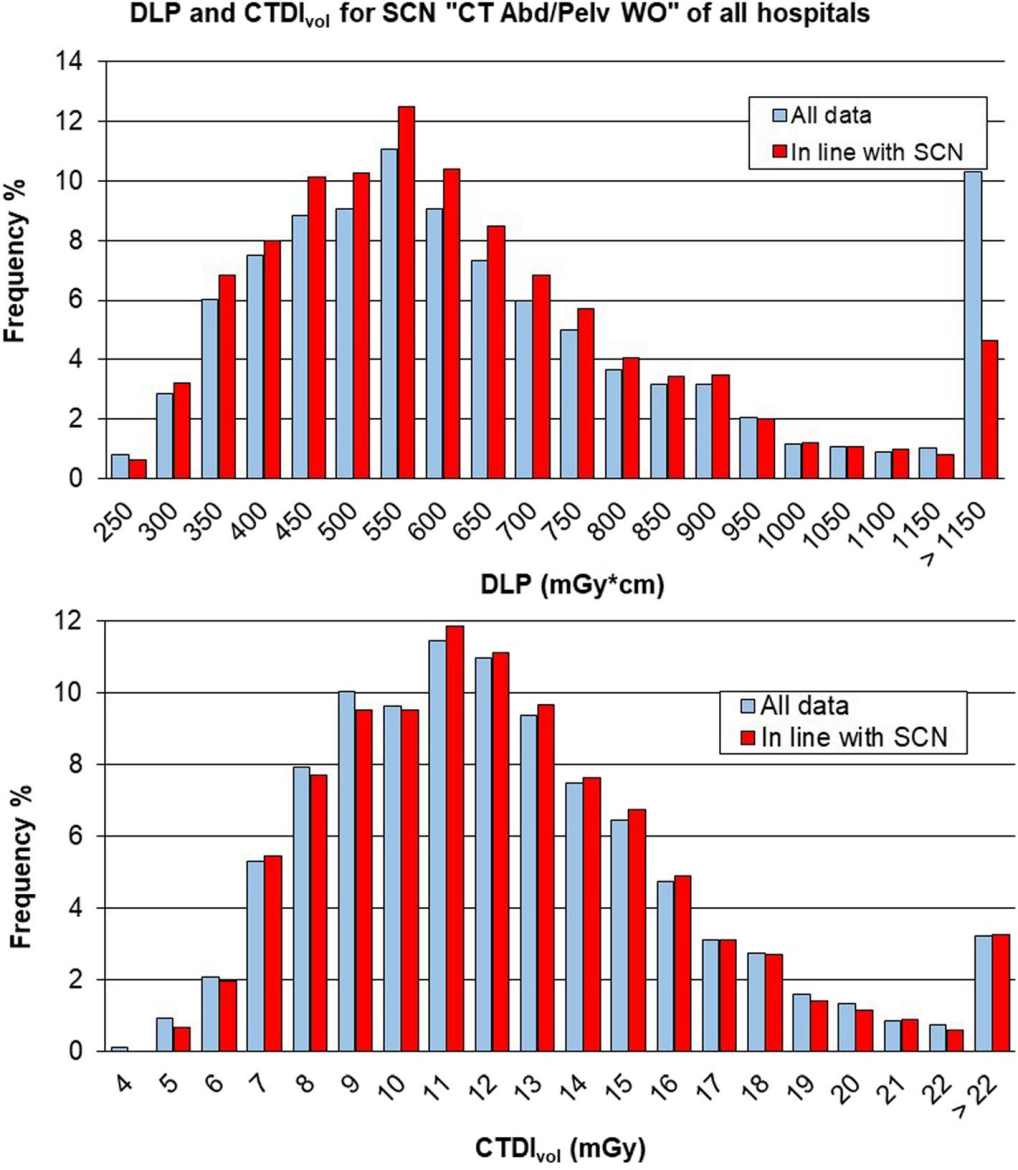
Comparison between distributions of total dose-length product (DLP) and volumetric computed tomography dose index (CTDI_vol_) of the whole data and those in line with the study common name (SCN) “CT Abd/Pelv WO”, i.e., studies with all series belonging to that cluster

The distributions of DLP and CTDI_vol_ obtained with these data are represented in Figs. 7, 8 and 9 in comparison with the previous overall distributions.

These comparisons are also summed up in Table 5. Even if the percentage of exams not in line with SCN for single hospitals reaches the 23%, the median values of dosimetric quantities for whole data vary for a few percent only, 7.7% at most (DLP of CT Head WO in Table 5).

**Table 5:** Dose-length product (DLP) and volumetric computed tomography dose index (CTDI_vol_) for “CT Head WO”, “CT Chest WO” and “CT Abd/Pelv WO”: data for exams in line with the study common name and for all exams

	DLP all hospitals (mGy × cm)			CTDI_vol_ all hospitals (mGy)			Number of exams
	Median	25th percentile	75th percentile	Median	25th percentile	75th percentile	
CT Head WO							
In line with SCN (A)	933	798	1022	58.6	54.2	58.9	25,252
All data (B)	1011	827	1024	57.8	53.3	58.9	26,965
Δ = (B - A)/B	+ 7.7%	+ 3.5%	+ 0.2%	-1.4%	-1.7%	0.0%	
CT Chest WO							
In line with SCN (A)	274	205	349	7.2	5.7	8.6	5,994
All data (B)	268	190	349	7.0	5.4	8.5	6,542
Δ = (B - A)/B	-2.2%	-7.9%	0.0%	-2.9%	-5.6%	-1.2%	
CT Abd/Pelv WO							
In line with SCN (A)	553	438	710	11.2	9.0	14.0	1,512
All data (B)	569	446	769	11.2	8.9	14.0	1,692
Δ = (B - A)/B	+ 2.8%	+ 1.8%	+ 7.7%	0.0%	-1.1%	0.0%	

## Discussion

The aim of this study was to evaluate advantages and disadvantages of a cloud-based CT dosimetric database, which represents the state of the art in terms of data collection for further optimisation.

These patient demographics and scan protocol information can be used for optimisation processes within each hospital and to compare the different facilities as well as for evaluation of risk due to patients’ exposure to ionising radiations, *e.g*., the distributions in terms of age and sex are necessary for a detailed risk analysis [1].

The dosimetric database was implemented overcoming the problems of dealing with hundreds of protocols from 13 CT scanners used in four hospitals with different RIS/PACS. Clustering through RadLex® playbook turned out to be a good choice for the subsequent analysis of data allowing data collection in a more homogeneous way. Our results show that by using RadLex® playbook, more than 400 CT procedures have been clustered in just 95 SCNs, but only ten SCNs described almost 80% of the exams. The prevalent exam is the “CT Head WO”, confirming the trend of other publications [1], which represents almost 40% of the studies. Despite the large use of CT in this anatomical region, these exposures are associated with a lower risk [21, 22].

As a preliminary analysis, the data for adult patients from the three main SCNs without the use of intravenous contrast agent were explored. The median values of DLP and CTDI_vol_ of all hospitals are well below the national DRL levels [18]. They are also close to European DRLs summarised by document 180 [19] and to the recent US Diagnostic Reference Levels and Achievable Doses [9] and Canadian Computed Tomography Survey [20]. The RDIM software was already in use in the hospitals even before 2017, and during these years, analysis was performed to allow optimisation of protocols, reducing patient exposure.

This preliminary analysis shows some differences between scanners of different hospitals as well, probably linked to the technology of the single devices, differences in protocol settings or specific requirements of the radiologists.

The presence of different RIS/PACS systems, CT scanners and protocols in each hospital, makes the implementation of this database more difficult and increases the probability of inaccuracies. A more detailed analysis of the single series present in the cloud highlights the presence of data that do not belong to the particular SCN. This can be explained by differences in the management of the reconciliation between the required exam and the performed one, due to clinical needs or emergencies. In particular, storage and medical reporting of neurologic examinations are organised in different ways. For example, in hospital 1, head CTs are completely disjoined by other anatomical regions.

However, variations in median values of DLP and CTDI_vol_ reach at most 8%. Because of the great amount of data, some inaccuracies were expected. Nevertheless, they do not heavily affect the dosimetric analysis obtained through the database, allowing the simpler use of the whole data without previous reviews.

In conclusion, the implementation of a dosimetric database requires considerable efforts to configure each scanner and to cluster the CT protocols of different hospitals; RadLex® playbook has proved to be an excellent tool for the comparison of homogeneous examinations. In this way, it is possible to optimise acquisitions obtaining a fair compromise between image quality and reduction of patient exposure. This process can be improved when several facilities, with expertise and skills in different clinical areas, populate the database and establish relevant benchmarks.

## Data Availability

The datasets used and analysed during the current study are available from the corresponding author on reasonable request.

## Abbreviations

CT: Computed tomography
CTDI_vol_: Volumetric computed tomography dose index
DICOM: Digital imaging and communications in medicine
DIR: Dose index registry
DLP: Dose-length product
DRL: Diagnostic reference level
RDIM: Radiation dose index monitoring
RTS: Radiation tracking software
SCN: Study common name
